# Carbonic Anhydrase Enhanced UV-Crosslinked PEG-DA/PEO Extruded Hydrogel Flexible Filaments and Durable Grids for CO_2_ Capture

**DOI:** 10.3390/gels9040341

**Published:** 2023-04-16

**Authors:** Jialong Shen, Sen Zhang, Xiaomeng Fang, Sonja Salmon

**Affiliations:** Department of Textile Engineering, Chemistry and Science, Wilson College of Textiles, North Carolina State University, Raleigh, NC 27695-8301, USA; jshen3@ncsu.edu (J.S.); szhang53@ncsu.edu (S.Z.)

**Keywords:** 3D printing, biocatalyst, carbonic anhydrase, CO_2_ capture, enzyme, hydrogel, immobilization, interpenetrating polymer network, reactive absorption, structured packing

## Abstract

In this study, poly (ethylene glycol) diacrylate/poly (ethylene oxide) (PEG-DA/PEO) interpenetrating polymer network hydrogels (IPNH) were extruded into 1D filaments and 2D grids. The suitability of this system for enzyme immobilization and CO_2_ capture application was validated. IPNH chemical composition was verified spectroscopically using FTIR. The extruded filament had an average tensile strength of 6.5 MPa and elongation at break of 80%. IPNH filament can be twisted and bent and therefore is suitable for further processing using conventional textile fabrication methods. Initial activity recovery of the entrapped carbonic anhydrase (CA) calculated from esterase activity, showed a decrease with an increase in enzyme dose, while activity retention of high enzyme dose samples was over 87% after 150 days of repeated washing and testing. IPNH 2D grids that were assembled into spiral roll structured packings exhibited increased CO_2_ capture efficiency with increasing enzyme dose. Long-term CO_2_ capture performance of the CA immobilized IPNH structured packing was tested in a continuous solvent recirculation experiment for 1032 h, where 52% of the initial CO_2_ capture performance and 34% of the enzyme contribution were retained. These results demonstrate the feasibility of using rapid UV-crosslinking to form enzyme-immobilized hydrogels by a geometrically-controllable extrusion process that uses analogous linear polymers for both viscosity enhancement and chain entanglement purposes, and achieves high activity retention and performance stability of the immobilized CA. Potential uses for this system extend to 3D printing inks and enzyme immobilization matrices for such diverse applications as biocatalytic reactors and biosensor fabrication.

## 1. Introduction

Anthropogenic CO_2_ emissions in the past half century have caused an abrupt increase in atmospheric CO_2_ levels [1] leading to an exacerbated greenhouse effect and global warming [2]. Meanwhile, population and GDP growth continue to spur increasing global total energy consumption, more than 80% of which is still represented by fossil fuels [3]. Therefore, eliminating CO_2_ emissions from large point sources, such as combustion-based power plants and energy-intensive hard-to-decarbonize industries, is among the most critical mitigations for combating the climate change crisis. For capturing CO_2_ at atmospheric pressure, the benchmark aqueous amine-based chemical absorption process is the most efficient, but its wide adoption is limited by the high energy requirement for solvent regeneration [4].

Enzyme-facilitated CO_2_ absorption using low-energy benign solvents, such as potassium carbonate-based solvents, has emerged as a promising alternative to the conventional amine-based solvents [5]. The enzyme responsible for accelerating CO_2_ absorption is called carbonic anhydrase (CA). CA is found in all domains of life where it catalyzes physiologically important reversible CO_2_ hydration and bicarbonate dehydration reactions with rates of up to one million substrate molecules per second per enzyme molecule [6]. Dissolved CA has exhibited remarkable CO_2_ absorption rate enhancement, but required regular replenishment to maintain high capture performance due to thermal deactivation of the free enzymes at elevated temperature conditions in the CO_2_ stripper [7]. Immobilization of CAs on physical supports or enzyme carriers [8,9,10,11] can stabilize CA against harsh solvents, facilitating reuse, and avoiding the high temperature environment by retaining CAs in the absorber column, which is typically maintained at much lower temperatures. Textile structured packing has recently been used as both a novel gas-liquid contactor and an enzyme immobilization support for CA [12], achieving synergistic enhancement of the CO_2_ capture efficiency using potassium carbonate-based and various other favorable solvents, including N,N-dimethylglycine (DMG), N-Methyldiethanolamine (MDEA), and sea water [13].

Additive manufacturing, or 3D printing, has recently emerged as a promising technology for integrating physical support fabrication together with enzyme immobilization [14,15,16]. 3D printing techniques that produce hydrogels, which can resemble natural enzymatic reaction environments, are especially suitable for direct enzyme inclusion by entrapment [15]. Among the first researchers in the field, Marquette and co-workers [17,18,19] used a digital light processing (DLP) technique to immobilize glucose oxidase (GOx), horseradish peroxidase (HRP), and alkaline phosphatase (ALP) in a poly(ethylene glycol) diacrylate (PEG-DA) hydrogel matrix with complex geometries for biosensing and tissue engineering applications. To fulfill the advantage of high resolution, DLP requires enzymes to be homogeneously dispersed throughout the photo-resin filling the entire reservoir, which is a far greater amount than the volume actually needed to fabricate the object, resulting in enzyme waste. Direct ink writing (DIW) is an extrusion-based printing technique that offers versatility and compatibility with practically any type of material as long as the formulated ink possesses the required rheological behaviors [20]. PEG-DA 700 (Mw~700), as a photo-crosslinkable oligomer, has relatively low viscosity unsuitable for DIW. Franzreb and co-workers [21,22] resolved this issue by adding viscosity enhancers such as colloidal clay and branched polysaccharides, resulting in printed hydrogels with tunable degradabilities. Lahann and co-workers [23] used high molecular weight poly (acrylic acid) (PAA) to modify the viscosity of the PEG-DA and obtained hydrogel fibers. Gao and co-workers [24,25] printed mechanically strong hydrogels from sodium alginate with acrylamide monomers, dual cross-linked by calcium cation and bis-acrylamide, respectively, and reinforced this with inorganic mineral particles for entrapment immobilization of GOx, catalase, and laccase. The so-called interpenetrating polymer networks (IPN) [26], formed by chain entanglements intertwining with cross-linked networks, were believed to be responsible for increased resistance against the propagation of cracks under stress [27].

**Figure 1:**
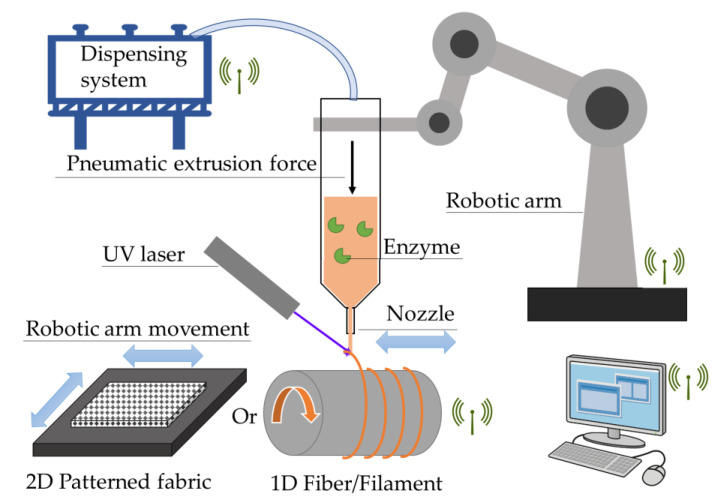
Hydrogel filament/grid fabrication system set-up.

In this study, we present a simple and effective method for printing PEG-DA hydrogels, in the form of monofilaments and 2D grids, for use in the direct entrapment immobilization of carbonic anhydrase, as an important first candidate, and other enzymes in the future (Figure 1). Syringe movement was controlled by a computer programmable robotic arm, and extrusion force was exerted by a pneumatic pump system. A glass stage or winding roller was used to collect the extruded 2D and 1D hydrogels, respectively.

**Figure 2:**
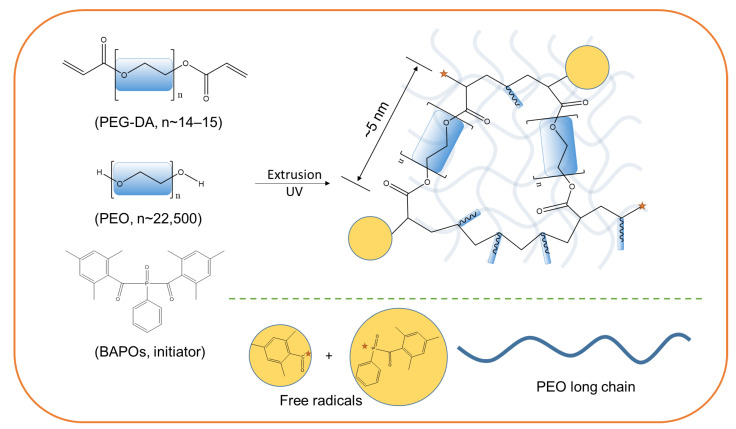
Schematics of the formation of PEG-DA/PEO interpenetrating polymer network hydrogel (IPNH). (Orange stars represent free redicals at the growing chain ends; blue rectangles represent ethylene oxide repeating units).

PEG-DA photo-curable resin was selected because it is well-known that the presence of poly (ethylene glycol) (PEG) around enzyme molecules provides stabilization effects against denaturing environments owing to the favorable interaction between the ether oxygen of the PEG and the enzyme outer surfaces [28,29,30,31]. The length of the PEG-DA 700 linkage (n of 14–15) in all-trans extended conformations is approximately 5 nm (Figure 2), commensurate with the diameter of a CA enzyme [32]. Therefore, the minimal possible pore size generated in this system will be able to accommodate the physical size of the CA molecule. High molecular weight polyethylene oxide (PEO), which is structurally analogous to the PEG repeating unit, was used for both viscosity enhancement and chain entanglement-inducing purposes, affording a fully compatible and extrudable ink that forms mechanically robust semi-IPN structures upon UV-curing (Figure 2). Semi-IPN structures are a type of IPN with a linear polymer composition in addition to a cross-linked polymer [26]. The extruded PEG-DA/PEO interpenetrating polymer network hydrogel (IPNH) filaments were evaluated for their tensile properties, flexibilities, and diameter as a function of extrusion pressure. Chemical composition and crosslinking reactions were confirmed spectroscopically using FTIR. Initial activity recovery and activity retention as a function of enzyme dose was tracked over 150 days by repeated washing and esterase activity assay in a 24-well plate. The printed IPNH 2D grids were assembled into cylindrical structured packings and CO_2_ capture efficiency as a function of enzyme dose was tested in a laboratory CO_2_ scrubber. Finally, the long-term CO_2_ capture performance was evaluated for 1032 h (43 days) in a continuous solvent recirculation experiment. The result of this study demonstrated the feasibility of using analogous linear polymers for both viscosity enhancement and chain entanglement purposes, achieving high activity retention and performance stability of the immobilized CA. This system shows potential for use as a 3D printing ink and enzyme immobilization matrix for diverse applications, such as biocatalytic reactors and biosensor fabrication.

## 2. Results and Discussion

### 2.1. Chemical Compositions of IPNHs

**Figure 3:**
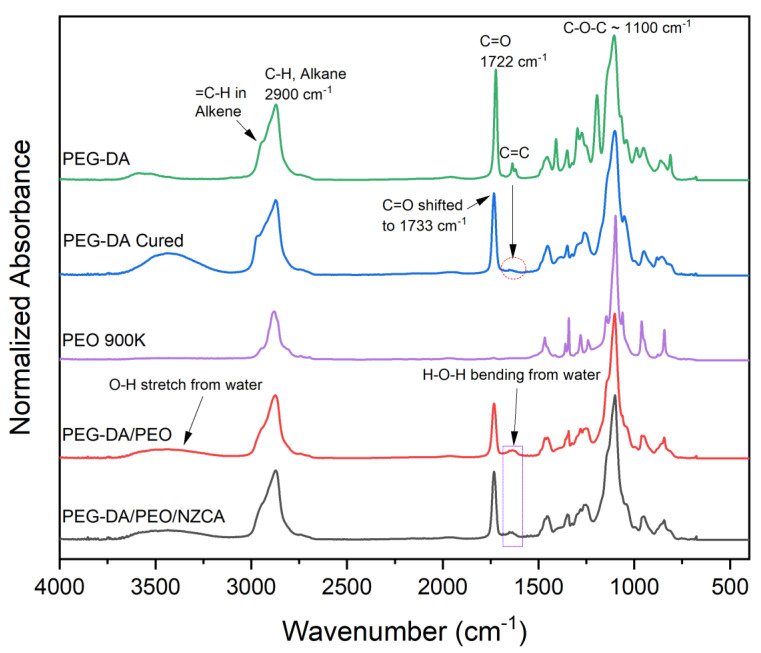
FTIR spectra of PEG-DA, PEG-DA after UV-cured, PEO 900K powder, extruded PEG-DA/PEO IPNH, and extruded PEG-DA/PEO IPNH with entrapped NZCA (From top to bottom).

The full list of chemical ingredients used to make the PEG-DA/PEO IPNH includes PEG-DA oligomer, PEO, water, and phenylbis (2,4,6-trimethylbenzoyl) phosphine oxide (BAPOs) photo-initiator in ethanol. Comparison of the FTIR spectra (not shown) of PEG-DA with and without BAPOs showed no visible differences due to low initiator concentration. As shown in Figure 3, uncured PEG-DA oligomer (top) has its highest peak at around 1100 cm^−1^ attributed to C-O-C stretching of the ether bonds in PEG repeating units [33]. Due to their structural similarity, this is also the strongest peak in PEO and PEG-DA/PEO IPNH samples. The next strongest peak appeared at around 1722 cm^−1^ as a result of the carbonyl bond (C=O) stretching from the acrylate end groups [34]. Additionally, C-H stretching of the saturated aliphatic methylene in the PEG repeating unit has a broad peak centered around 2900 cm^−1^, with a shoulder at slightly over 3000 cm^−1^, which is instead assigned to the C-H stretching vibration of the uncured alkene (=C-H) [35]. The characteristic peak for uncured acrylate arises from the C=C double bond stretching appearing as a doublet at around 1636 cm^−1^. After UV-curing, this doublet diminished to a very low intensity residual hump, indicating the conversion of double bond. It is also noticeable that the carbonyl bond (C=O) stretching shifted from 1722 to 1733 cm^−1^ due to the polymerization of the PEG-DA oligomer. Because there are three =C-H bonds for each un-cured alkene, the shoulder located in the C-H stretching region above 3000 cm^−1^ does not disappear. This indicates that, the double bond conversion reaction does not go to completion. This is reasonable because the polymerization reaction is so rapid that the vitrification of the hydrogel happens almost instantaneously upon UV irradiation. The vitrified crosslinked network impeded the diffusion of the radicals and prevented further reaction with some of the un-reacted double bonds [36]. Due to the presence of un-reacted double bonds, the pore (or cage) size of the IPNH can have a wide distribution permitting the entrapment of not only single enzyme molecules, but also enzyme aggregates commonly formed at high enzyme concentrations. Russo et al. [37] reported the formation of enzyme aggregates in potassium carbonate solvent at a CA concentration of >0.3 g/L and confirmed that the CA aggregates remained active and acted as a heterogeneous catalyst. This feature of the PEG-DA/PEO IPNH may prove to be useful for retaining larger enzyme loading in the form of active enzyme aggregates. The addition of aqueous PEO solution at a ratio of 6:4 (*v/v*, 10 *w/v*% PEO solution: PEG-DA) in the ink formulation did not significantly change the curing reaction as evidenced by the similar peak profiles between cured PEG-DA and PEG-DA/PEO IPNH within the 1200–1500 cm^−1^ region. The doublet at around 1636 cm^−1^ also disappeared and the shoulder at around 3000 cm^−1^ became less prominent, presumably due to the dilution effect from the added PEO which contributed to the higher intensity of the main saturated C-H stretching peak. Chemically, enzymes are long chains of polymerized amino acids that have the strongest peaks at 1634 cm^−1^ corresponding to amide I band (C=O stretching) and at 1540 cm^−1^ corresponding to amide II band (N-H bending and C-N stretching) [13]. However, no significant difference in FTIR spectra can be observed between samples of PEG-DA/PEO IPNH with and without entrapped NZCA enzyme (Figure 3). This is partly due to signal overlapping caused by the water H-O-H bending peak [38] in the same region around 1640 cm^−1^ [39]. In addition, low overall enzyme concentration and homogenous distribution of it throughout the entire volume of the IPNH, rather than concentrated on the surface, could make the amide I signal too weak to observe. Magnified spectra of PEG-DA/PEO IPNH with and without entrapped NZCA for the regions between 1000 and 2000 cm^−1^ are included in the (Supplementary Information Figure S1) along with the NZCA enzyme spectrum for comparison. Due to the H-O-H bending of adsorbed water, the difference in peak intensity at 1634 cm^−1^ was insufficient to conclusively detect the presence of enzyme, even though the enzyme is known to be present by the additive nature of the fabrication process. However, two new peaks emerged for the IPNH with the enzyme present at 1583 cm^−1^ and 1518 cm^−1^ in the conventional amide II band region. Because amide II is sensitive to protein interactions with each other and with its surroundings [40], peaks in that region can be a result of the NZCA enzyme interacting with ethylene oxide repeating units of the polymer network and water through, e.g., hydrogen bonding. Acknowledging these shortcomings in IR detection of entrapped enzymes, the presence of the NZCA enzyme was verified using other methods, namely, an esterase activity assay and tests of CO_2_ capture efficiency enhancement, as described in detail in later sections.

### 2.2. PEG-DA/PEO IPNH Filament

**Figure 4:**
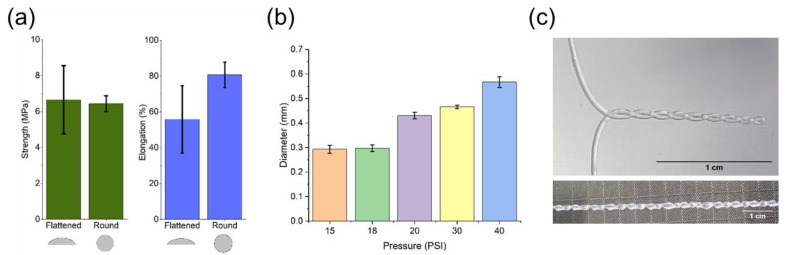
Properties of extruded PEG-DA/PEO IPNH filament: (**a**) Effect of curing on a surface or in air on the tensile strength and elongation of filaments extruded at 20 psi, (**b**) Effect of extrusion pressure on the diameter of filaments cured in air, and (**c**) Photos demonstrating the flexibility of a filament, which can be bent and twisted (Scale bars = 1 cm).

To produce a filament, the extruded IPNH ink can be cured instantly in air before coming in contact with the roller or after being deposited on a collector surface, depending on the incident angle of the UV laser. Filament cross-sections manifest distinctive geometries depending on the curing and depositing sequence. The cure-in-air method produces round shape filaments while the cure-on-surface filament is essentially half-oval shape. The average tensile strengths of the filaments cured by the two methods are almost identical at around 6.5 MPa, with a higher standard deviation for the flattened one (Figure 4a). This tensile strength value compares favorably with that of high-strength hydrogels summarized in a recent review [41]. For example, poly (vinyl alcohol) hydrogel reinforced by hydroxyapatite and tannic acid only had a tensile strength of 0.43 MPa [42]. A type of advanced high performance hydrogel related to IPNH is called a double network hydrogel (DNH) [43], which has literature reported tensile strength values falling mostly in the range of 1–10 MPa [41]. On the other hand, the average elongation at break of the round shape filament is higher, at around 80%, and with a lower standard deviation compared to that of the flattened filament. This percent elongation value sits at the lower end of the literature reported range of 80–2000% [41]. For our structured packing application, the hydrogel material needs to stay in place, therefore a large deformation is not necessarily an advantage. As shown in Figure 4b, the diameter of the extruded filament can be varied by adjusting the extrusion pressure controlled by the pneumatic pump. The flexibility of the IPNH filament, including bending and twisting, is excellent as demonstrated in Figure 4c, and it is suitable for future additional processing such as weaving and braiding.

### 2.3. PEG-DA/PEO IPNH Grid and Structured Packing

**Figure 5:**
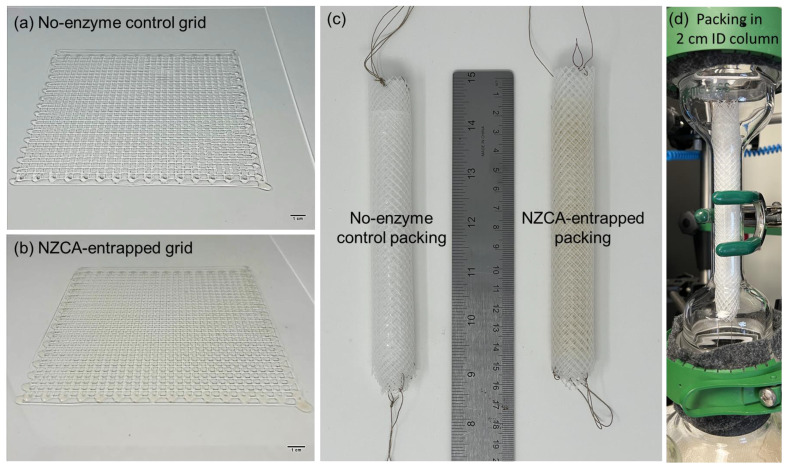
Extruded PEG-DA/PEO IPNH grids: (**a**) No-enzyme control (Scale bar = 1 cm), (**b**) NZCA-entrapped (Scale bar = 1 cm), (**c**) Assembled into 2 cm O.D. structured packing, and (**d**) Assembled packing fitted in a 2 cm I.D. CO_2_ absorption column.

One of the advantages of 3D printing, versus weaving or braiding, is its rapid prototyping capability through computer aided design (CAD). In Figure 5, photos of a printed 2D IPNH grid structure as well as that with an entrapped NZCA enzyme are shown. The color of the NZCA-entrapped grid comes from the natural color of the enzyme solution while the structure of the printed grid was not affected by the addition of enzyme solution as shown in Figure 5b. Printed grids were rolled up, along with a layer of nylon mesh that acted as a catalytically inert structural support and spacer, into a final spiral wound structured packing design [12] with a length of 15 cm and an outer diameter (O.D.) of 2 cm (Figure 5c), which fit snuggly in the laboratory absorption column shown in Figure 5d. The installed structured packing was then tested for CO_2_ capture efficiency, and the results are discussed in detail in later sections.

### 2.4. Esterase Activity Assay of PEG-DA/PEO IPNH Grid

**Figure 6:**
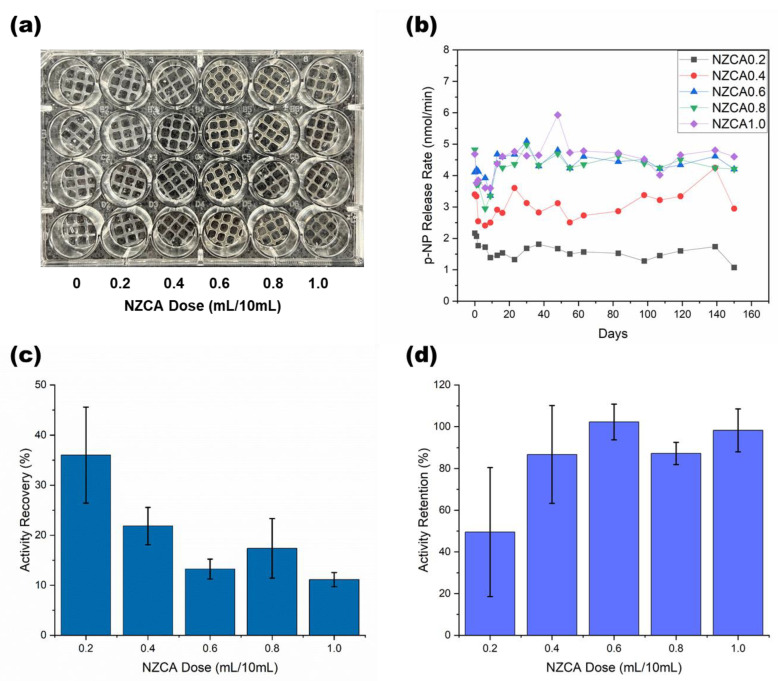
Enzyme activity assay on extruded PEG-DA/PEO IPNH grids with different NZCA doses: (**a**) Samples are cut into circles that fit in the wells of a 24-well plate, (**b**) Long-term stability of the immobilized NZCA over 150 days in buffer and at room temperature subject to repeated wash and testing, (**c**) Activity recovery of the immobilized NZCA on Day 0, calculated against the activity of a same amount of dissolved NZCA used in the immobilization, (**d**) Activity retention on Day 150.

Due to limitations in FTIR sensitivity, additional confirmation of the presence and functionality of NZCA enzymes in the PEG-DA/PEO IPNH was needed. Since enzymes catalyze very specific chemical reactions, an assay that detects changes in reaction product concentration can be used to determine the amount of active enzyme present. To eliminate the potential effect of UV exposure as a variable in IPNH enzyme activity measurements, a control experiment evaluating the effect of UV-irradiation time on dissolved enzyme activity was carried out for 0, 30, 60, 120, and 240 s. More than 98% activity was retained after 240 s irradiation when exposing a pH 7.2 aqueous solution of NZCA (starting activity of 3.3 nmol/min, similar to the IPNHs) at a distance of 15 cm from the UV light source (Supplementary Information Figure S2). This evidence indicates that UV-irradiation during the normal curing process conditions (<30 s, 15 cm distance) does not significantly alter enzyme activity and any large changes in activity detected later should be attributed to other factors. In order to carry out activity assays on immobilized NZCA, samples of the printed IPNH grids with and without entrapped NZCA were cut into circular shapes that fit in the wells of a 24-well assay plate used to measure the UV-vis absorbance of the product molecules (Figure 6a). PEG-DA/PEO IPNH without entrapped NZCA (dose = 0) was used as a control and its signals were subtracted from all other samples as the background. The esterase activity assay was run on the same samples repeatedly over a total of 150 days, with multiple rinsing steps using buffers in between each consecutive test (Figure 6b). The ranking of the initial activity of samples with different enzyme doses generally corresponded to the amount of the enzyme dose, with an exception observed at the highest enzyme dose samples, where 0.8 and 1.0 (mL/10 mL) had very similar absolute activities (Day 0 data shown in Figure 6b). Sample activities dropped steadily for the initial few days, followed by a noticeable upturn, especially for higher enzyme dose samples, occurring at around 6–9 days. During incubation in the aqueous environment, rearrangement of potential enzyme aggregates is more likely at higher enzyme doses, which could explain this improved activity effect. From Day 10 and onwards, the high enzyme dose samples (NZCA 0.6–1.0) maintained steady activity until the end of the tests.

When the initial detected activities were compared against the activity of the same amount of dissolved NZCA used to make each immobilized sample, the percentage is termed “activity recovery”, which is commonly used for evaluating the efficiency of the enzyme immobilization procedure. As shown in Figure 6c, an average of more than 35% of the enzyme activity added was detected in the immobilized sample with the lowest enzyme dose (NZCA 0.2). However, as the enzyme dose increased, lower activity recovery percentages were detected. Because the immobilization was an additive entrapment technique, where all the enzyme mixed into the system was retained during fabrication, the low activity recovery percentage is an indication that enzyme aggregation may have occurred that prevented esterase assay substrate from reaching all enzyme active sites. As recognized in the literature, activity recovery is not a monotonic function of the enzyme concentration and there exists a saturation concentration beyond which the activity recovery decreases [44]. The differences in activity recovery between NZCA 0.2 and the group NZCA 0.4–1.0 were associated with calculated *p*-values between 0.0005–0.047, which indicate highly significant differences, meaning that a saturation concentration could reside between the 0.2 and 0.4 doses. On the other hand, differences within the groups NZCA 0.4–1.0 had *p*-values between 0.18 and 0.98, signifying insignificant differences and similar underlying physical mechanisms. See the (Supporting Information Table S1) for a complete list of comparison pairs.

Another notable point is that while activity recovery seeks to evaluate the efficiency of enzyme incorporation during material fabrication it does not necessarily account for the absolute activity or the final performance of immobilized enzymes. For example, as shown in Figure 6d, the higher enzyme dose samples retained more than 87% of their initial activity on the last day of the test. In this case, the formation of enzyme aggregates and the slow activation of those “buried” activities over a long period of time may be beneficial. In fact, this potential for “controlled exposure/release” of “fresh” enzyme activity from within an immobilization matrix could have significant practical value, and represents a useful hybrid of conventional immobilized and dissolved enzyme approaches that is somewhat analogous to controlled release concepts in drug delivery. Statistical significance analysis showed that while the activity retentions among the groups NZCA 0.4–1.0 were largely similar, with *p*-values 0.84–1 (Supporting Information, Table S2, includes a complete list of comparison pairs), the NZCA 0.2 group had lower average activity retention and larger sample-to-sample variations, indicated by the error bar in Figure 6d. This emphasizes the beneficial effects that can arise from enzyme aggregate formation at higher enzyme loading, generating samples with high activity retention and low sample-to-sample variations.

The NZCA-IPNH activity retention (>87%) measured at higher enzyme doses compares well to other enzyme immobilization systems reported in the literature. Cui et al. [45] immobilized catalase on metal organic frameworks (MOFs) and encapsulated them in large mesoporous silica to obtain 81% activity recovery and 50% activity retention after 10 cycles of testing. Park et al. [46] immobilized lysozyme as crosslinked enzyme aggregates (CLEAs) on electrospun chitosan nanofibers, which retained 75% of the initial activity after 80 days of storage at room temperature. Ren et al. [47] embedded carbonic anhydrase in MOFs via co-precipitation which were in turn encapsulated in poly(vinyl alcohol) (PVA)/chitosan composite hydrogel networks. After 11 cycles of repeated washing (three times with Milli-Q water between each cycle) and reuse, the hydrogel membrane maintained 50% of its original activity. Sahoo et al. [48] immobilized CA through covalent linkage between amine groups of the enzyme and epoxy functionalized micro-flower-like inorganic support and found a retention of 90% of initial activity after 15 cycles of reuse. The high activity retention can be attributed to the stabilization effect of the chemical linkages. Hou et al. [49] functionalized a TiO_2_ nanoparticle surface using 3-amino-propyltriethoxysilane (APTES) salinization followed by glutaraldehyde activation and CA enzyme covalent immobilization. After 20 cycles of reuse (washing with Tris buffer and recovery by filtration after each use), 85% of initial activity was retained. These comparisons highlight that the NZCA-IPNH technique achieves activity retention on par with that of CAs immobilized by covalent methods, without the need to use special chemical crosslinkers for improving stability. In addition, delayed exposure of enzyme active sites from enzyme aggregates over long time periods (19 cycles, over 150 days) may explain the sustained activity in NZCA-IPNH samples without the help of protein-specific covalent crosslinkers. Exposing NZCA-IPNH to multiple washing cycles during incubation over long time periods was conducted to simulate some of the conditions these materials could encounter in an industrial CO_2_ scrubbing application, such as constant immersion in aqueous solutions.

### 2.5. Laboratory CO_2_ Scrubber Test of PEG-DA/PEO IPNH Structured Packing

**Figure 7:**
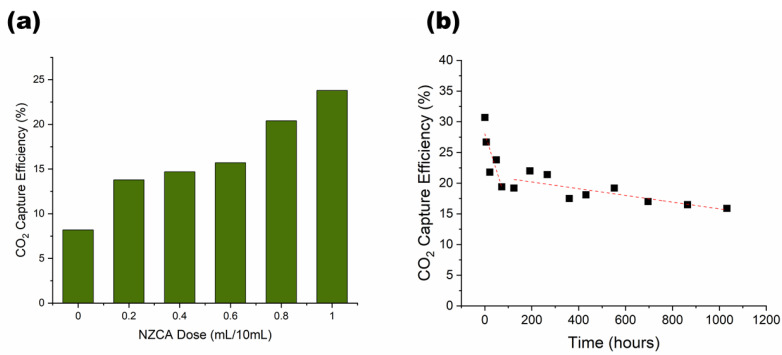
CO_2_ gas scrubber test of the assembled PEG-DA/PEO IPNH structured packings: CO_2_ capture efficiency (**a**) as a function of NZCA dose and (**b**) over 1032 h of continuous solvent recirculation.

To evaluate their suitability for CO_2_ capture applications, assembled IPNH structured packings, made with varying enzyme doses, were installed and tested in the absorption column of a laboratory CO_2_ scrubber (Figure 5d). As shown in Figure 7a, CO_2_ capture performance of the no enzyme control packing (NZCA = 0) had only 8% capture efficiency, while increasing enzyme dose gradually increased the capture efficiency to 24% at an enzyme dose of 1 mL/10 mL. This performance corresponds to an enzyme enhancement factor of three, which compares favorably with literature reported values of 1.1–3.6 for packed column absorbers using immobilized CA [6]. For further comparison, Fabbricino et al. [50] immobilized CA by “in vivo” anchoring of the enzyme on cell membranes to create dispersed cell debris that acted as biocatalysts with an enhancement factor of 1.3–2.4. Leimbrink et al. [51] tested immobilized CA in the form of buoyant micro-particles supplied by Akermin Inc. (St. Louis, MO, USA) in 30% MDEA solvent in a counter current packed column and found a six-fold enhancement in CO_2_ absorption with the immobilized CA. Previously, we reported enhancement factors of 2 and 2.9 for NZCA immobilized on textile structured packing, using chitosan entrapment and surface covalent attachment methods, respectively, compared to the no-enzyme bare textile structured packing [12,13]. Note that the enhancement factors we reported for the textile packings and the NZCA-IPNH packings exclude the contributions by the bare packing, which itself can offer significant improvement compared to conventional random or structured packings [12] owing to superior liquid transport properties of hydrophilic yarn structures [52]. Therefore, the total enhancement factor of NZCA-IPNH packings compared to conventional packing materials could be even higher.

In Figure 7a, the “sluggish” increase in CO_2_ capture efficiency when enzyme loading increases from NZCA 0.2 to NZCA 0.6 is because apparent % activity recovery decreased with increased enzyme loading, especially exhibiting a large precipitous drop between 0.2 and 0.4, as shown in Figure 6c, indicating diminished enzyme active site accessibility. In other words, results indicate that some enzymes loaded into the IPNH fibers are buried too deep inside the hydrogel to come in contact with CO_2_ substrate. CA is a diffusion limited enzyme, meaning that CA can catalyze the conversion of CO_2_ to bicarbonate faster than the rate at which CO_2_ molecules diffuse into its active site, this mass transfer limitation leads to the observation that catalytic enhancements for CO_2_ absorption are dominated by the presence of enzymes near the surface of the physical support and may appear relatively insensitive to increased enzyme loading in the bulk of the material. Nevertheless, at sufficiently high bulk enzyme loading, which the esterase activity assay does a better job identifying [49,53], more enzyme molecules are expected to reside near the surface, resulting in higher CO_2_ capture efficiencies. Based on the results, strategies to enhance CO_2_ absorption by IPNH fibers could include optimizing current IPNH recipes to promote enzyme availability at surface interfaces, producing fibers with smaller diameters or rough surfaces (higher relative surface area), and producing core-shell extruded fibers, where CA is primarily or only included in the outer shell to enhance its exposure during CO_2_ capture applications. Future studies could also include closer examination of enzyme aggregation phenomena and the role this plays in biocatalyst stability and performance.

The main incentives for using immobilized enzymes in industrial applications are their ease of recovery and reuse. Therefore, the activity lifetime or longevity is an especially important metric for evaluating the potential of an immobilized enzyme product for commercialization. An assay-scale longevity test like the one described in Section 2.4 is the most commonly used methodology to evaluate longevity. However, the reaction conditions used during assay-scale tests are not exactly the same as what the sample will really experience in the final application, especially in dynamic conditions, such as constantly flowing solvent. Therefore, an additional and important way to test the longevity of the NZCA immobilized IPNH structured packing is to subject it to continuous solvent flow over a long period of time. As shown in Figure 7b, the capture efficiency of the NZCA immobilized IPNH structured packing experienced a rapid drop in the first 100 h of operation and then a much slower steady drop over the later period of the test. The initial performance drop is probably related to an initial burst leaching of partially immobilized surface enzyme, after which a slower enzyme leaching from the inner volume of the hydrogel occurs over a longer period of time. This is in contrast to the esterase activity results, which indicated a reversal of the activity drop after 6–9 days and a steady activity retention over the next 4–5 months for high enzyme dose samples. That can be explained by the differences in reaction time scale of the two tests: the esterase activity test is completed in 30–60 min while the CO_2_ hydration reaction runs on a time scale of seconds. Therefore, the esterase activity assay is not limited by the time it takes for substrate molecules to diffuse through the hydrogel and is able to reach (detect) enzymes entrapped deep inside the hydrogel, while the CO_2_ hydration reaction in the scrubber test is diffusion-limited and only enzymes on the most outer surface are effective for rate enhancement. Nevertheless, even after 1032 h (43 days) of continuous use, while exposed to constantly flowing alkaline aqueous liquid, 52% of the initial CO_2_ capture performance and 34% of the enzyme contribution were retained—a very encouraging result.

## 3. Conclusions

In this study, we demonstrated the extrusion of PEG-DA/PEO interpenetrating polymer network hydrogels (IPNHs) into 1D filaments and 2D grids and evaluated their suitability for enzyme immobilization and application for CO_2_ capture. Chemical composition and crosslinking reactions were confirmed spectroscopically using FTIR. The filament tensile strength compares favorably with that of other double network hydrogels (DNHs) reported in the literature, and its elongation at break sits at the lower end of the normal range for DNH, which can be favorable for certain applications. The IPNH filament can be twisted and bent and is therefore suitable for further processing using conventional textile fabrication methods. Although, initial activity recovery of the entrapped NZCA, calculated by an esterase activity assay, decreased with increasing enzyme dose, the activity retentions of high enzyme dose samples were over 87% after 150 days of repeated incubation, washing and testing. The IPNH 2D grids were assembled into spiral roll structured packings and their CO_2_ capture efficiency increased with increasing amount of immobilized enzyme. Long-term CO_2_ capture performance of the NZCA immobilized IPNH structured packing was tested in a continuous solvent recirculation experiment for 1032 h, where 52% of the initial CO_2_ capture performance and 34% of the enzyme contribution were retained. The results of this study demonstrate the feasibility of using a UV-crosslinked system of analogous linear polymers for both viscosity enhancement and chain entanglement purposes to achieve high activity retention and performance stability of entrapped immobilized CA. This system shows good potential for use as a 3D printing ink and enzyme immobilization matrix for diverse applications such as biocatalytic reactors and biosensor fabrication.

## 4. Materials and Methods

### 4.1. Materials

Potassium carbonate (K_2_CO_3_), potassium bicarbonate (KHCO_3_), 4-nitrophenol (p-NP), 4-nitrophenyl acetate (p-NPAc), 1N hydrochloric acid, and absolute ethanol, were purchased from Fisher Scientific (Waltham, MA, USA). Trizma^®^ base, poly (ethylene oxide) (PEO, Mv~900,000), poly(ethylene glycol) diacrylate (PEG-DA, Mn~700), phenylbis(2,4,6-trimethylbenzoyl)phosphine oxide (BAPOs) were purchased from Millipore-Sigma (Burlington, MA, USA). All chemicals were used as received, without further purification. Experimental thermostable microbial carbonic anhydrase (CA) with esterase activity of 13.5 U/mL was obtained from Novozymes A/S, Bagsvaerd, Denmark, and referred to as NZCA stock enzyme solution in this study.

### 4.2. Preparation of Extrusion Solutions

High molecular weight PEO (Mv~900,000) was dissolved in deionized water at 10 wt% concentration and used as a stock solution. BAPOs was dissolved in absolute ethanol at a concentration of 10 mg/mL as the initiator stock solution and stored in the refrigerator until use. For no enzyme IPNH, 6 g of stock PEO solution was first mixed with 4 mL of PEG-DA in a plastic jar using a planetary mixer (DAC 330-100 SE, FlackTek Speed Mixer, Landrum, SC, USA) at 3000 RPM for 1 min, then 0.5 mL of stock initiator solution was added to the mixture and mixed for another 1 min at 3000 RPM. For enzyme entrapped IPNH, 6 g of stock PEO solution was first mixed with 0.2–1.0 mL of stock enzyme solution for 2 min at 3000 RPM, allowing PEO chains to fully interact with enzyme molecules and serve as a compatibilizer for the next step where PEG-DA was added and mixed for 1 min at 3000 RPM. Mixing stock enzyme solution with PEO separately in the first step is necessary as mixing all three ingredients at the same time resulted in enzyme precipitation. Finally, 0.5 mL of stock initiator was added and mixed for 1 min.

### 4.3. Extrusion Procedures

Extrusion solution prepared in Section 4.2 was loaded into a syringe that was compatible with the pneumatic pump dispensing system (Nordson EFD Ultimus V, Nordson Corporation, Westlake, TX, USA) and connected to a 20 gauge (I.D. = 0.026″, O.D. = 0.036″ length = 2″) blunt needle through a luer lock mechanism. Extrusion pressure was varied from 15 to 40 psi to study the effect of pressure on filament diameter and was kept at 20 psi for filaments made for mechanical testing. Dymax BlueWave^®^ 200 V3.0 (Torrington, CT, USA), which emits light with wavelength of 280–450 nm and has a power output of 40 W/cm^2^, was used for the UV curing process. The cure-in-air method requires the UV laser to be pointed at the solution jet right after leaving the needle and before touching the collector. The cure-on-surface method cures the extruded solution after it has been laid on the collector. Next, 2D grids were printed using the movement of a robotic arm (M1, Dobot Robotics, Shenzhen, China) programmed using the accompanying M1 studio v1.5.3 software. The grid was 15 cm × 15 cm with a 0.5 cm line path spacing. The extrusion pressure was 35–40 psi for the 2D grid and the robotic arm movement speed was set at 7%.

### 4.4. Tensile Testing

Filaments were air dried at room temperature and cut into 15 cm specimens. Each sample was tested and averaged over at least 5 specimens using an MTS system (MTS-30G, MTS Systems Corporation, Eden Prairie, MN, USA). The gauge length was set at 10 cm and the crosshead speed was 0.5 cm/min.

### 4.5. Fourier-Transform Infrared Spectroscopy (FTIR)

A Nicolet Nexus 470 spectrometer was used to collect FTIR spectra of PEG-DA as-received liquid sample, UV-cured PEG-DA, PEO powder, PEG-DA/PEO IPNH, and PEG-DA/PEO IPNH with entrapped NZCA enzymes. The hydrogel samples were naturally air dried at ambient conditions before testing. The spectrometer was equipped with a Nicolet OMNI germanium crystal Attenuated Total Reflection (ATR) sampling head and a total of 64 scans were collected for each sample at a resolution of 4 cm^−1^.

### 4.6. Esterase Activity Assay

Adaption of a conventional esterase activity assay for testing immobilized CA has been described in detail elsewhere [12]. Briefly, IPNH grid samples with or without entrapped NZCA were cut into circle shapes with an outer diameter of 5/8″ that fit in the wells of a 24-well plate. Four replicates were included for each immobilized sample. Tris buffer 25 mM and pH 7.2 was used as both a washing and assay buffer. Assay buffer (950 μL) and 50 μL of 8 mM pNPAc substrate were added to each well containing a circular IPNH grid. A TECAN Spark UV-VIS spectrophotometer was used to measure the change in the absorbance of the p-NP generated by the CA catalyzed hydrolysis of p-NPAc over a period of 30 min for a total of 60 measurements for each well. A calibration curve was made that related the O.D. of the p-NP absorbance to known concentrations of this molecule. p-NP release rates were then calculated for each sample. Samples were repeatedly washed and tested without leaving the assay plate. Between two consecutive assay runs, liquid was replaced with fresh buffer two times before finally being replaced with the assay buffer. Incubations, washing and assay tests were carried out at ambient temperature (~22 °C).

### 4.7. Laboratory CO_2_ Gas Scrubber Test

CO_2_ capture efficiencies of IPNH structured packings were evaluated in a laboratory CO_2_ gas scrubber. A schematic of the scrubber set-up is available in Ref. [13]. K_2_CO_3_ solvent (10 wt%), prepared with K_2_CO_3_/KHCO_3_ w/w ratio of 85/15 and final pH of 10.5, was used as the CO_2_ absorption solvent. A solvent flow rate of 120 mL/min and gas flow rate of 1 L/min were used as the standard CO_2_ scrubber testing conditions. Tests were carried out at ambient temperature. The gas mixture contained 10 vol% CO_2_ made by mixing 0.1 L/min CO_2_ and 0.9 L/min N_2_ from tanks controlled by mass flow controllers. CO_2_ concentration was measured from a dried split stream from the gas mixture exiting the absorption column. CO_2_ capture efficiency was calculated by Equation (1) using a stable baseline reading before turning on the liquid flow, and after the scrubber reached a steady state capture.

(1) $$CO_{2}\;capture\;efficiency\;\left(\%\right)=\frac{CO_{2\;before}-CO_{2\;after}}{CO_{2\;before}}\times 100\%,$$

The continuous solvent recirculation experiment was carried out at room temperature using 10 wt% K_2_CO_3_ to evaluate the long-term operational stability of the packing. Solvent flowed through the IPNH structured packing during the entire 1032 h. The standard scrubber testing conditions mentioned above were used to periodically measure the CO_2_ capture efficiency at different time intervals using fresh lean solvent for each measurement.

## Data Availability

The data presented in this study are available on request from the corresponding author.

## Supplementary Materials

The following supporting information can be downloaded at: https://www.mdpi.com/article/10.3390/gels9040341/s1, Figure S1: FTIR spectra of PEG-DA/PEO IPNH, PEG-DA/PEO IPNH with entrapped NZCA enzyme, and NZCA enzyme; Figure S2: Effect of UV irradiation on dissolved NZCA enzyme activity; Table S1: Statistical significance tests among immobilized NZCA of different enzyme doses based on activity recovery on day 0; Table S2: Statistical significance tests among immobilized NZCA of different enzyme doses based on activity retention on day 150.

## References

[1] Lindsey R. Climate Change: Atmospheric Carbon Dioxide. https://www.climate.gov/news-features/understanding-climate/climate-change-atmospheric-carbon-dioxide.

[2] Lenton T., Rockström J., Gaffney O., Rahmstorf S., Richardson K., Steffen W., Shellnhuber H.J. (2019). Climate Tipping Points—Too Risky to Bet Against. Nature.

[3] IEA Greenhouse Gas Emissions from Energy: Overview. https://www.iea.org/reports/greenhouse-gas-emissions-from-energy-overview.

[4] Bettenhausen C.A. (2021). Carbon Capture’s Steep Climb. Chem. Eng. News.

[5] Reardon J., Bucholz T., Hulvey M., Tuttle J., Shaffer A., Pulvirenti D., Weber L., Killian K., Zaks A. (2014). Low Energy CO_2_ Capture Enabled by Biocatalyst Delivery System. Energy Procedia.

[6] Salmon S., House A. (2015). Enzyme-Catalyzed Solvents for CO_2_ Separation. Novel Materials for Carbon Dioxide Mitigation Technology.

[7] Qi G., Liu K., House A., Salmon S., Ambedkar B., Frimpong R.A., Remias J.E., Liu K. (2018). Laboratory to Bench-Scale Evaluation of an Integrated CO_2_ Capture System Using a Thermostable Carbonic Anhydrase Promoted K_2_CO_3_ Solvent with Low Temperature Vacuum Stripping. Appl. Energy.

[8] Molina-Fernández C., Luis P. (2021). Immobilization of Carbonic Anhydrase for CO_2_ Capture and Its Industrial Implementation: A Review. J. CO2 Util..

[9] Shen J., Salmon S. (2023). Biocatalytic Membranes for Carbon Capture and Utilization. Membranes.

[10] Rasouli H., Nguyen K., Iliuta M.C. (2022). Recent Advancements in Carbonic Anhydrase Immobilization and Its Implementation in CO_2_ Capture Technologies: A Review. Sep. Purif. Technol..

[11] Russo M.E., Capasso C., Marzocchella A., Salatino P. (2022). Immobilization of Carbonic Anhydrase for CO_2_ Capture and Utilization. Appl. Microbiol. Biotechnol..

[12] Shen J., Yuan Y., Salmon S. (2022). Carbonic Anhydrase Immobilized on Textile Structured Packing Using Chitosan Entrapment for CO_2_ Capture. ACS Sustain. Chem. Eng..

[13] Shen J., Yuan Y., Salmon S. (2022). Durable and Versatile Immobilized Carbonic Anhydrase on Textile Structured Packing for CO_2_ Capture. Catalysts.

[14] Shao Y., Liao Z., Gao B., He B. (2022). Emerging 3D Printing Strategies for Enzyme Immobilization: Materials, Methods, and Applications. ACS Omega.

[15] Shen J., Zhang S., Fang X., Salmon S. (2022). Advances in 3D Gel Printing for Enzyme Immobilization. Gels.

[16] Pose-Boirazian T., Martínez-Costas J., Eibes G. (2022). 3D Printing: An Emerging Technology for Biocatalyst Immobilization. Macromol. Biosci..

[17] Devillard C.D., Mandon C.A., Lambert S.A., Blum L.J., Marquette C.A. (2018). Bioinspired Multi-Activities 4D Printing Objects: A New Approach Toward Complex Tissue Engineering. Biotechnol. J..

[18] Mandon C.A., Blum L.J., Marquette C.A. (2017). 3D-4D Printed Objects: New Bioactive Material Opportunities. Micromachines.

[19] Mandon C.A., Blum L.J., Marquette C.A. (2016). Adding Biomolecular Recognition Capability to 3D Printed Objects. Anal. Chem..

[20] Saadi M.A.S.R., Maguire A., Pottackal N., Thakur M.S.H., Ikram M.M., Hart A.J., Ajayan P.M., Rahman M.M. (2022). Direct Ink Writing: A 3D Printing Technology for Diverse Materials. Adv. Mater..

[21] Schmieg B., Schimek A., Franzreb M. (2018). Development and Performance of a 3D-Printable Poly(Ethylene Glycol) Diacrylate Hydrogel Suitable for Enzyme Entrapment and Long-Term Biocatalytic Applications. Eng. Life Sci..

[22] Schmieg B., Döbber J., Kirschhöfer F., Pohl M., Franzreb M. (2019). Advantages of Hydrogel-Based 3D-Printed Enzyme Reactors and Their Limitations for Biocatalysis. Front. Bioeng. Biotechnol..

[23] Steier A., Schmieg B., Irtel von Brenndorff Y., Meier M., Nirschl H., Franzreb M., Lahann J. (2020). Enzyme Scaffolds with Hierarchically Defined Properties via 3D Jet Writing. Macromol. Biosci..

[24] Shen X., Yang M., Cui C., Cao H. (2019). In Situ Immobilization of Glucose Oxidase and Catalase in a Hybrid Interpenetrating Polymer Network by 3D Bioprinting and Its Application. Colloids Surfaces A Physicochem. Eng. Asp..

[25] Liu J., Shen X., Zheng Z., Li M., Zhu X., Cao H., Cui C. (2020). Immobilization of Laccase by 3D Bioprinting and Its Application in the Biodegradation of Phenolic Compounds. Int. J. Biol. Macromol..

[26] Sperling L.H., Hu R. (2014). Interpenetrating Polymer Networks. Polymer Blends Handbook.

[27] Farooq U., Teuwen J., Dransfeld C. (2020). Toughening of Epoxy Systems with Interpenetrating Polymer Network (IPN): A Review. Polymers.

[28] Wang X., Bowman J., Tu S., Nykypanchuk D., Kuksenok O., Minko S. (2021). Polyethylene Glycol Crowder’s Effect on Enzyme Aggregation, Thermal Stability, and Residual Catalytic Activity. Langmuir.

[29] Chapman R., Stenzel M.H. (2019). All Wrapped up: Stabilization of Enzymes within Single Enzyme Nanoparticles. J. Am. Chem. Soc..

[30] Pérez B., Coletta A., Pedersen J.N., Petersen S.V., Periole X., Pedersen J.S., Sessions R.B., Guo Z., Perriman A., Schiøtt B. (2018). Insight into the Molecular Mechanism behind PEG-Mediated Stabilization of Biofluid Lipases. Sci. Rep..

[31] Yang Z., Domach M., Auger R., Yang F.X., Russell A.J. (1996). Polyethylene Glycol-Induced Stabilization of Subtilisin. Enzyme Microb. Technol..

[32] Krishnamurthy V.M., Kaufman G.K., Urbach A.R., Gitlin I., Gudiksen K.L., Weibel D.B., Whitesides G.M. (2008). Carbonic Anhydrase as a Model for Biophysical and Physical-Organic Studies of Proteins and Protein−Ligand Binding. Chem. Rev..

[33] Pramono E., Utomo S.B., Wulandari V., Clegg F. (2016). FTIR Studies on the Effect of Concentration of Polyethylene Glycol on Polimerization of Shellac. J. Phys. Conf. Ser..

[34] Askari F., Zandi M., Shokrolahi P., Tabatabaei M.H., Hajirasoliha E. (2019). Reduction in Protein Absorption on Ophthalmic Lenses by PEGDA Bulk Modification of Silicone Acrylate-Based Formulation. Prog. Biomater..

[35] Worzakowska M. (2017). TG/DSC/FTIR/QMS Studies on the Oxidative Decomposition of Terpene Acrylate Homopolymers. J. Therm. Anal. Calorim..

[36] Herrera-González A.M., Caldera-Villalobos M., Pérez-Mondragón A.A., Cuevas-Suárez C.E., González-López J.A. (2019). Analysis of Double Bond Conversion of Photopolymerizable Monomers by FTIR-ATR Spectroscopy. J. Chem. Educ..

[37] Peirce S., Perfetto R., Russo M.E., Capasso C., Rossi M., Salatino P., Marzocchella A. (2018). Characterization of Technical Grade Carbonic Anhydrase as Biocatalyst for CO_2_ Capture in Potassium Carbonate Solutions. Greenh. Gases Sci. Technol..

[38] Seki T., Chiang K.Y., Yu C.C., Yu X., Okuno M., Hunger J., Nagata Y., Bonn M. (2020). The Bending Mode of Water: A Powerful Probe for Hydrogen Bond Structure of Aqueous Systems. J. Phys. Chem. Lett..

[39] Medders G.R., Paesani F. (2015). Infrared and Raman Spectroscopy of Liquid Water through “First-Principles” Many-Body Molecular Dynamics. J. Chem. Theory Comput..

[40] Sadat A., Joye I.J. (2020). Peak Fitting Applied to Fourier Transform Infrared and Raman Spectroscopic Analysis of Proteins. Appl. Sci..

[41] Hua J., Ng P.F., Fei B. (2018). High-Strength Hydrogels: Microstructure Design, Characterization and Applications. J. Polym. Sci. Part B Polym. Phys..

[42] Xiang C., Zhang X., Zhang J., Chen W., Li X., Wei X., Li P. (2022). A Porous Hydrogel with High Mechanical Strength and Biocompatibility for Bone Tissue Engineering. J. Funct. Biomater..

[43] Ji D., Kim J. (2021). Recent Strategies for Strengthening and Stiffening Tough Hydrogels. Adv. NanoBiomed Res..

[44] Sun J., Wang C., Wang Y., Ji S., Liu W. (2019). Immobilization of Carbonic Anhydrase on Polyethylenimine/Dopamine Codeposited Membranes. J. Appl. Polym. Sci..

[45] Cui J., Feng Y., Jia S. (2018). Silica Encapsulated Catalase@metal-Organic Framework Composite: A Highly Stable and Recyclable Biocatalyst. Chem. Eng. J..

[46] Park J.-M., Kim M., Park H.-S., Jang A., Min J., Kim Y.-H. (2013). Immobilization of Lysozyme-CLEA onto Electrospun Chitosan Nanofiber for Effective Antibacterial Applications. Int. J. Biol. Macromol..

[47] Ren S., Li C., Tan Z., Hou Y., Jia S., Cui J. (2019). Carbonic Anhydrase@ZIF-8 Hydrogel Composite Membrane with Improved Recycling and Stability for Efficient CO_2_ Capture. J. Agric. Food Chem..

[48] Sahoo P.C., Kumar M., Singh A., Singh M.P., Puri S.K., Ramakumar S.S.V. (2017). Accelerated CO_2_ Capture in Hybrid Solvent Using Co-Immobilized Enzyme/Complex on a Hetero-Functionalized Support. J. CO2 Util..

[49] Hou J., Dong G., Xiao B., Malassigne C., Chen V. (2015). Preparation of Titania Based Biocatalytic Nanoparticles and Membranes for CO_2_ Conversion. J. Mater. Chem. A.

[50] Fabbricino S., Del Prete S., Russo M.E., Capasso C., Marzocchella A., Salatino P. (2021). In Vivo Immobilized Carbonic Anhydrase and Its Effect on the Enhancement of CO_2_ Absorption Rate. J. Biotechnol..

[51] Leimbrink M., Nikoleit K.G., Spitzer R., Salmon S., Bucholz T., Górak A., Skiborowski M. (2018). Enzymatic Reactive Absorption of CO_2_ in MDEA by Means of an Innovative Biocatalyst Delivery System. Chem. Eng. J..

[52] Yuan Y., Zhang Y., Bilheux H., Salmon S. (2021). Biocatalytic Yarn for Peroxide Decomposition with Controlled Liquid Transport. Adv. Mater. Interfaces.

[53] Hou J., Ji C., Dong G., Xiao B., Ye Y., Chen V. (2015). Biocatalytic Janus Membranes for CO_2_ Removal Utilizing Carbonic Anhydrase. J. Mater. Chem. A.

