# Supplementary material for: Carbonic Anhydrase Enhanced UV-Crosslinked PEG-DA/PEO Extruded Hydrogel Flexible Filaments and Durable Grids for CO2 Capture
Source: Gels. 2023 Apr 16;9(4):341. doi: 10.3390/gels9040341 (PMC10137505; doi:10.3390/gels9040341)
Supplement: Supplementary file 1 [file gels-09-00341-s001.zip › gels-2343818-supplementary.pdf]

Carbonic Anhydrase Enhanced UV-Crosslinked PEG-DA/PEO Extruded Hydrogel Flexible Filaments and Durable Grids for CO_2 Capture

Jialong Shen, Sen Zhang, Xiaomeng Fang* and Sonja Salmon*

Department of Textile Engineering, Chemistry and Science, Wilson College of Textiles, North Carolina State University, Raleigh, NC 27695-8301, USA; jshen3@ncsu.edu (J.S.); szhang53@ncsu.edu (S.Z.)

*Correspondence: xfang3@ncsu.edu (X.F.); sisalmon@ncsu.edu (S.S.)

Supplementary Information

Contains:

2 Figures and 2 Tables

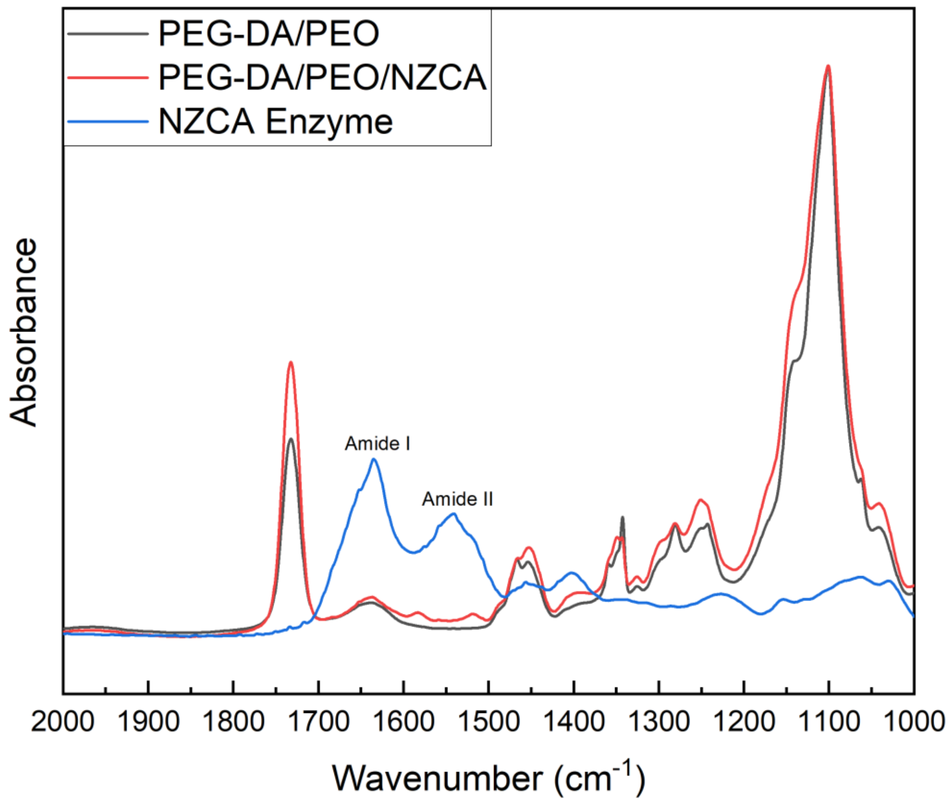

Figure S1. FTIR spectra of PEG-DA/PEO IPNH, PEG-DA/PEO IPNH with entrapped NZCA enzyme, and NZCA enzyme.

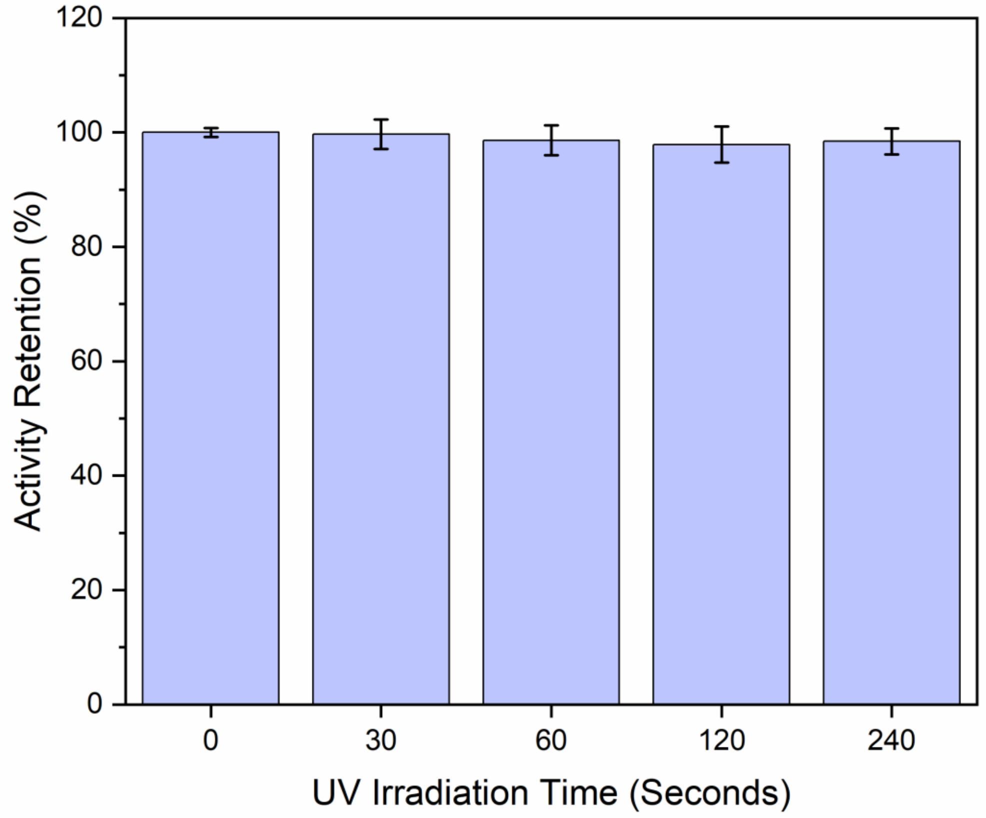

Figure S2. Effect of UV irradiation on dissolved NZCA enzyme activity.

Table S1. Statistical significance tests among immobilized NZCA of difference enzyme doses based on activity recovery on day 0.

Comparison pair	p.adj
NZCA0.4-NZCA0.2	0.0470324
NZCA0.6-NZCA0.2	0.0012406
NZCA0.8-NZCA0.2	0.0071350
NZCA1.0-NZCA0.2	0.0005196
NZCA0.6-NZCA0.4	0.3620395
NZCA0.8-NZCA0.4	0.8593567
NZCA1.0-NZCA0.4	0.1801902
NZCA0.8-NZCA0.6	0.8882249
NZCA1.0-NZCA0.6	0.9892495
NZCA1.0-NZCA0.8	0.6497923

Table S2. Statistical significance tests among immobilized NZCA of difference enzyme doses based on activity retention on day 150.

Comparison pair	p.adj
NZCA0.4-NZCA0.2	0.1524637
NZCA0.6-NZCA0.2	0.0232981
NZCA0.8-NZCA0.2	0.1439779
NZCA1.0-NZCA0.2	0.0388347
NZCA0.6-NZCA0.4	0.8377666
NZCA0.8-NZCA0.4	0.9999997
NZCA1.0-NZCA0.4	0.9377288
NZCA0.8-NZCA0.6	0.8532056
NZCA1.0-NZCA0.6	0.9987245
NZCA1.0-NZCA0.8	0.9467189
